# Elevated pretreatment neutrophil-to-lymphocyte ratio indicate low survival rate in apatinib-treated patients with non-small cell lung cancer: A STROBE-compliant article

**DOI:** 10.1097/MD.0000000000032043

**Published:** 2022-11-25

**Authors:** Ya-Nan Wan, Hai-Ming Chen, Xin-Fu Liu, Wei-Guang Gu, Yi-Yu Lu

**Affiliations:** a Department of Internal Medicine, Nanhai Sixth People’s Hospital, Foshan City, China; b Department of Oncology, The Sixth Affiliated Hospital, South China University of Technology, Foshan City, China; c Department of Oncology, Nanhai People’s Hospital, Foshan City, China; d Department of Oncology and Hematology, Shaoyang Central Hospital, Shaoyang, China.

**Keywords:** drug resistance, multiline treatment, NLR, OS, PFS, survival

## Abstract

This study aimed to analyze the predictive value of the neutrophil-to-lymphocyte ratio (NLR) to better clarify which patients with advanced non-small cell lung cancer (NSCLC) would benefit most from apatinib after multiline treatment for drug resistance. This observational cohort study involved patients with advanced NSCLC who were treated with apatinib between May 2016 to May 2018. The participants in this study had previously been treated with at least two treatment regimens. Multivariate logistic regression and Cox proportional risk models were used to evaluate the overall survival (OS) and progression-free survival (PFS) of the pretreatment NLR. A total of 125 patients were reviewed. The median age was 64 years (range, 33–92); and 32.8% of the patients were female. Only 0.8% of the patients had an Eastern Cooperative Oncology Group Performance Status (ECOG-PS) score ≥ 2. In multivariate analysis, pretreatment NLR ≥ 5 had an independent correlation with inferior OS (median 2.07 vs 3.40 months; HR 1.493, 95% CI 1.022–2.182; *P* = .038) and inferior PFS (median 1.83 vs 2.76 months; HR 1.478, 95% CI 1.015–2.153; *P* = .042). Elevated pretreatment NLR is associated with shorter OS and PFS in patients with advanced NSCLC treated with apatinib after multiline treatment for drug resistance.

## 1. Introduction

Despite recent improvements in strategies for diagnosing and treating lung cancer, it remains one of the main reasons for cancer-associated deaths globally, making it necessary to find more appropriate and effective treatments for patients with non-small cell lung cancer (NSCLC). A study recently showed that tumors require angiogenesis to spread to other tissues.^[1]^ Tumor-secreted vascular endothelial growth factor (VEGF) and other growth factors induce angiogenesis, which indicate anti-angiogenesis therapy is one of the hot spots of current research.^[1]^

Apatinib is a small molecule inhibitor, which is highly selective in competing with the ATP binding site of intracellular VEGF receptor-2, blocking the downstream signal transduction, inhibiting the activity of intracellular VEGF-2, and inhibiting tumor angiogenesis, thus playing an anti-tumor role.^[2–4]^ Clinical studies showed that it had potent anticancer activity in lung, liver, stomach, ovarian, and soft tissue cancers, and other malignant tumors.^[5–10]^ However, we found that the efficacy of apatinib varied widely among patients. Some patients had satisfactory results, while others had no benefit from apatinib treatment in the same pathologic type of tumor. Apatinib was widely used in a variety of tumors because of its broad antitumor activity, which allowed oncologists to use it without detecting any markers. Hence, it is imperative to identify markers that can help screen suitable population and predict their prognoses.

Inflammation plays an indispensable role in the evolution of cancers, mainly by promoting angiogenesis, cancer cell proliferation, and tumor metastasis, thus inhibiting anti-tumor effects. With increase in systemic inflammation, the prognosis of these patients tends to be worse. Neutrophilic leukocytosis in peripheral blood reflects cancer inflammation and was considered a prognostic immune biomarker for various malignant tumors. In recent years, biomarkers extracted from peripheral blood, such as the ratio of platelets-to-albumin, lymphocytes-to-monocytes and neutrophil-to-lymphocyte ratio, have attracted more attention. The neutrophil/lymphocyte ratio (NLR) derived from these newly identified noninvasive biomarkers, whose accessibility and predictive ability for advanced malignant tumors have been sufficiently validated and reported in previous clinical studies, including breast, stomach, esophagus, and colorectal cancer.^[11–16]^ Besides, in recent years, NLR has been used to explore the survival prognosis of patients with limited advanced NSCLC. NLR seems to be a prognostic indicator for chemotherapy, immunotherapy, and targeted therapy in these patients.^[17–20]^

However, only few studies showed the relationship between NLR and survival prognoses of patients with advanced NSCLC and who are receiving apatinib. Therefore, this retrospective study aimed to compare the apatinib-treated efficacy of patients with advanced NSCLC at different levels of pretreatment NLR after multiple treatment for drug resistance.

## 2. Material and Methods

### 2.1. Patients

This observational cohort study retrospectively collected clinical treatment history and laboratory data from apatinib-treated patients with advanced cancer in a tertiary hospital. A total of 125 patients were recruited for this study from May 2016 to May 2018. The study data obtained from electronic medical records and clinical examination reporting included: patients’ basic information, Eastern Cooperative Oncology Group (ECOG) stage, smoking status, drinking status, pathological type, clinical stage, and blood indices. Patients who did not have laboratory tests at the beginning of the apatinib therapy and who underwent chemotherapy, radiation therapy, chemoradiotherapy or targeted therapy three weeks before laboratory examination, were excluded. Moreover, patients with inaccurate or incomplete clinical data or those who were lost to follow-up were excluded. Patients with related diseases that can cause abnormal NLR or those with inadequate liver or renal function, tumor emergency, or other severe primary disease, were excluded for this study.

Patients who were eligible for the study were hospitalized after primary histopathological analysis and imaging examination confirmed tumor progression. Patients with secondary treatment failure were diagnosed with stage III or IV NSCLC, including locally-advanced tumors that were difficult to remove and those that invaded adjacent structures or other distant organs. All patients who had never received apatinib treatment prior to the present regimen and had received more than two previous treatment regimens were included.

All patients had complete clinical data before receiving apatinib. Qualified patients were anonymized, and their clinical data were obtained and counted from the electronic medical record system. The study was conducted in accordance with the Declaration of Helsinki (as revised in 2000). The study was approved by the Research Ethics Committee of Hospital (NO.: [2018] 1) and was registered in Chinese Clinical Trials. All subjects were fully informed and signed the informed consent form before the study.

### 2.2. Treatment assessment

Clinicians and radiologists jointly assessed and classified the response according to the Response Evaluation Criteria in Solid Tumours (RECIST 1.1 criteria^[21]^). At the same time, adverse events were assessed using the Common Terminology Criteria for Adverse Events (CTCAE) 4.0 during treatment.

### 2.3. Definitions of variables

This study reflected the host inflammatory marker NLR. The NLR was calculated by measuring absolute neutrophils and lymphocytes in the peripheral blood before apatinib treatment began. Overall survival (OS) was counted from starting treatment with apatinib to death, and progression-free survival (PFS) was counted from starting treatment with apatinib to clinical or radiographic progression or death. After NLR grouping of the cohort using the above threshold values, PFS and OS were analyzed after the apatinib treatment to determine whether these markers were related to PFS and OS.

### 2.4. Statistical analysis

We analyzed the clinical characteristics of enrolled patients with NSCLC satisfying the inclusion criteria. The receiver operating characteristic curve (ROC curve) was drawn and the area under curve was calculated to determine the prediction accuracy and critical values of NLR. The categorical variables were described as counts and percentages, while the continuous data were expressed as median and range. Baseline clinical characteristics of the high and low NLR groups were compared using the χ^2^ test. The Cox proportional risk model was used to count the risk ratio (HR) and 95% confidence interval (CI) to evaluate factors potentially affecting PFS and OS. Univariate and multivariate analyses were used to identify critical independent prognostic factors. The Kaplan–Meier method was used to draw survival curves to analyze OS and PFS, and the log-rank test was utilized to compare the difference between pairs of data variables. A *P* value < .05 was considered statistically significant. The above data were analyzed using SPSS 23. 0 software (Chicagos).

## 3. Results

### 3.1. Patient characteristics

We included a total of 125 patients with advanced NSCLC treated with apatinib, two of whom were excluded due to incomplete data and loss to follow-up (n = 2). A total of 125 patients with advanced cancer were analyzed in this study, and their demographic characteristics were shown in Table 1. All patients had ≥ 2 prior systemic treatment regimens. Median follow-up was 2.77 months (range: 0.30–29.20 months), and all patients died from disease progression. The median age of patients in the study was 64 years (33–92 years), wherein 32.8% of these patients were female. There were 103 nonsmokers (82.4%) and 22 smokers (17.6%). Patients with metastasis were present in 100 (80.0%) patients. NLR data were missing in two patients.

**Table 1:** Patient characteristics (n = 125).

Characteristic	N (%)
Age (yr)	
Median	64.0
Range	33.0–92.0
<65	63 (50.4%)
≥65	62 (49.6%)
BMI (kg/m^2^)	
<18.5	20 (16.0%)
≥18.5, <24	91 (72.8%)
≥24	14 (11.2%)
Gender	
Male	84 (67.2%)
Female	41 (32.8%)
ECOG PS	
0	64 (51.2%)
1	60 (48.0%)
≥2	1 (0.8%)
Prior lines of therapy	
2	41 (32.8%)
3	15 (12.0%)
≥4	69 (55.2%)
Smoking history	
Smoker	22 (17.6%)
Nonsmoker	103 (82.4%)
Alcohol drinking	
Drinker	12 (9.6%)
Nondrinker	113 (90.4%)
Metastasis	
Yes	100 (80.0%)
No	2520.0%)
NLR	
Low NLR	50 (40.0%)
High NLR	75 (60.0%)

BMI = body mass index, ECOG PS = Eastern Cooperative Oncology Group Performance Status, NLR = neutrophil-to-lymphocyte ratio.

### 3.2. Cutoff value of NLR

**Figure 1.**
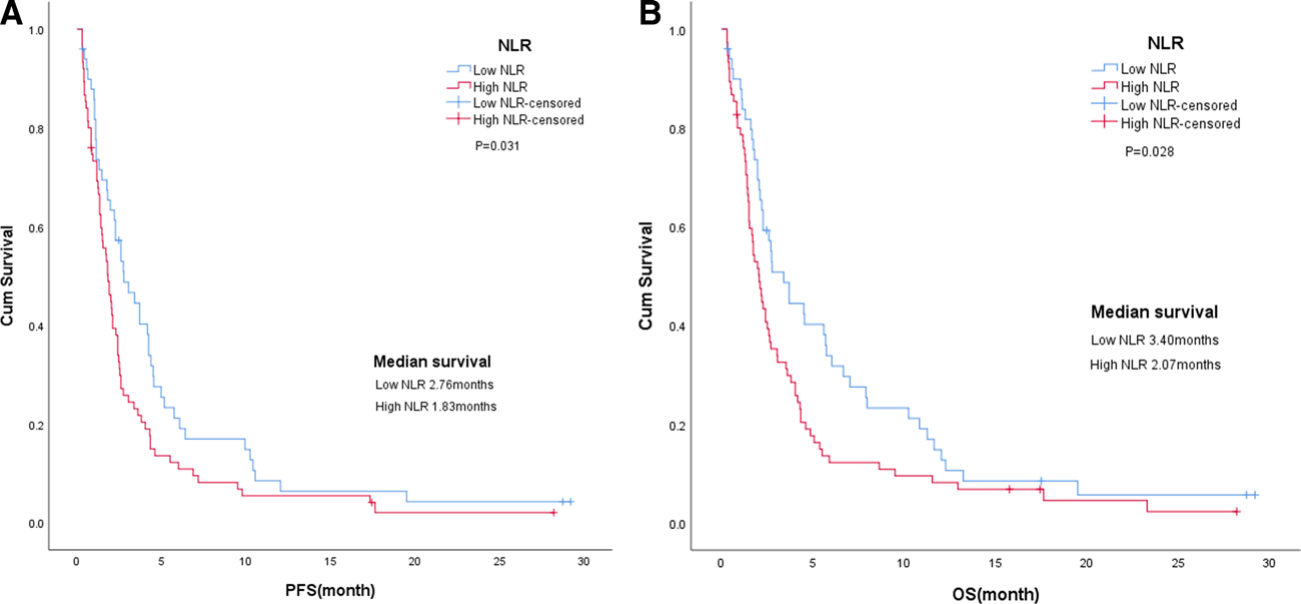
The receiver operating characteristic curve (ROC) for the neutrophil/lymphocyte ratio (NLR).

NLR was taken as the test variable and the ROC curve was drawn to determine the cutoff value. The area under the curve was 0.507. The cutoff value of the NLR was 3.72 with a sensitivity of 67.8% and a specificity of 57.1% (Fig. 1). Median pretreatment NLR was 4.26 (range 0.39–75.20), including NLR < 3.72 (40.0%) in 50 patients and NLR ≥ 3.72 (60.0%) in 75 patients.

### 3.3. Survival analyses

**Figure 2.**
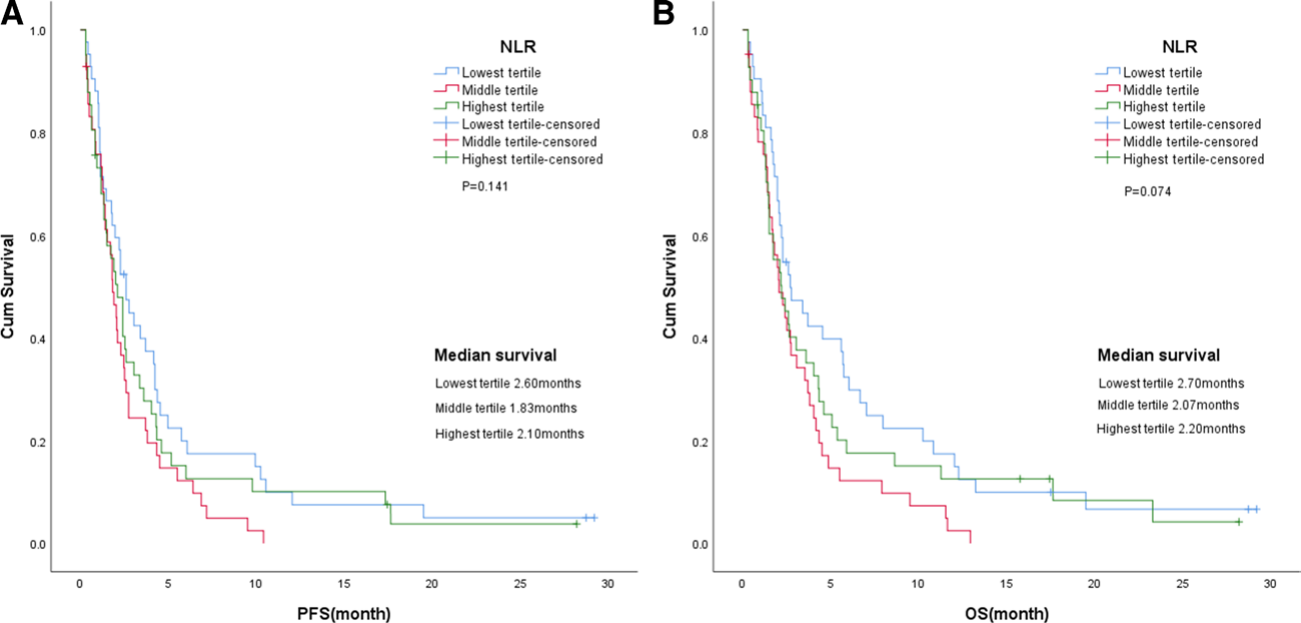
Kaplan–Meier survival curves of progression-free survival and overall survival in patients with advanced non-small cell lung cancer (NSCLC). (A) Progression-free survival (PFS) according to the NLR. (B) Overall survival (OS) according to the NLR. NLR = neutrophil/lymphocyte ratio.

**Figure 3.**
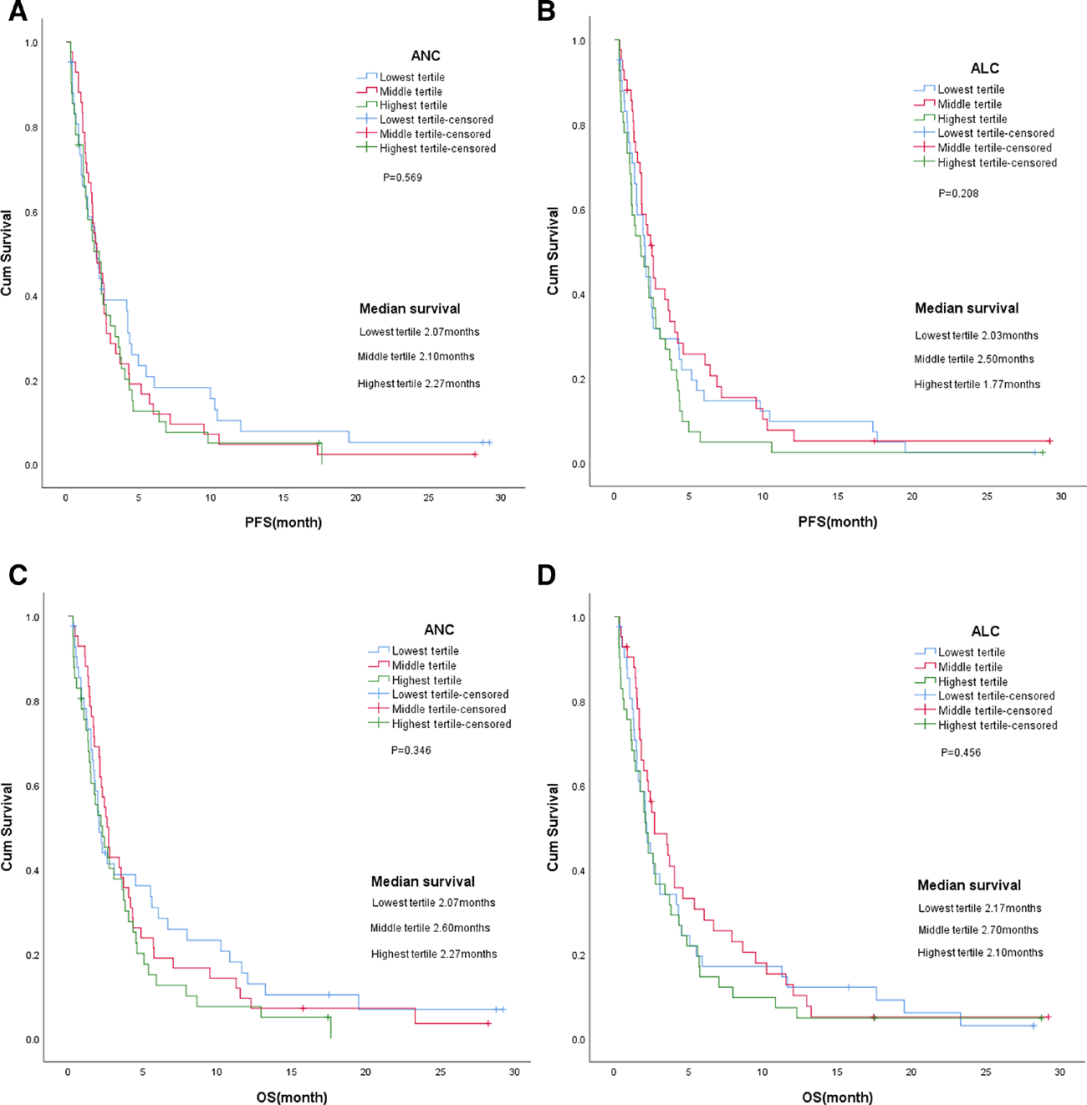
Kaplan–Meier survival curves of progression-free survival (A) and overall survival (B) according to NLR tertiles. NLR = neutrophil/lymphocyte ratio.

Correlation analysis showed that there were no statistically significant differences in age, gender, ECOG, body mass index, line of treatment, history of smoking and alcohol drinking, and metastasis between the high and low NLR groups (Table 2). Among the analysis group, higher pretreatment NLR was associated with worse OS and PFS (Fig. 2, Tables 3 and 4). In the univariate analysis, high NLR was associated with inferior OS and PFS compared with low NLR (median 2.07 vs 3.40 months; HR 1.523, 95% CI 1.043–2.215, *P* = .029 and median 1.83 vs 2.76 months; HR 1.501, 95% CI 1.033–2.182, *P* = .029, respectively; Table 3). When we divided the NLR into 3 groups, the cohort showed a significant difference in OS and PFS among the groups (Fig. 3). The final multivariate model included gender and NLR. In the multivariate analysis, only high NLR was associated with inferior OS and PFS (HR 1.493, 95% CI 1.022–2.182, *P* = .038 and HR 1.478, 95% CI 1.015–2.153, *P* = .042, respectively; Table 4).

**Table 2:** Comparison of related factors between low level and high level NLR groups.

Characteristics	NLR		*P* value	χ/*t* value
	Low NLR	High NLR		
Gender				
Male	36	48	.351	0.871
Female	14	27		
Age				
<65	30	33	.080	3.072
≥65	20	42		
BMI (kg/m^2^)				
<18.5	7	13	.671	0.799
≥18.5, <24	36	55		
≥24	7	7		
ECOG-PS				
0	20	44	.060	5.643
1	29	31		
≥2	1	0		
Lines of treatment				
2	16	25	.854	0.316
3	7	8		
≥4	27	42		
Smoke history				
Yes	10	12	.565	0.331
No	40	63		
Alcohol drinking				
No	45	68	.901	0.015
Yes	5	7		
Metastasis				
Yes	41	59	.648	0.208
No	9	16		

BMI = body mass index, ECOG PS = Eastern Cooperative Oncology Group Performance Status, NLR = neutrophil-to-lymphocyte ratio.

**Table 3:** The progression-free survival and overall survival in univariate analysis.

Characteristics	N	PFS		OS	
		HR (95% CI)	*P* value	HR (95% CI)	*P* value
Gender					
Male	84	0.553 (0.367, 0.834)	.005	0.625 (0.419, 0.933)	.021
Female	41				
Age					
<65	63	1.196 (0.832, 1.718)	.334	1.224 (0.849, 1.763)	.279
≥65	62				
BMI					
<18.5	20	0.869 (0.629, 1.200)	.393	0.836 (0.602, 1.160)	.284
≥18.5, <24	97				
≥24	14				
ECOG-PS					
0	64	0.768 (0.535, 1.102)	.152	0.706 (0.489, 1.018)	.062
1	60				
≥2	1				
Lines of treatment					
2	41	0.868 (0.709, 1.064)	.174	0.839 (0.685, 1.028)	.091
3	15				
≥4	69				
Smoke history					
Yes	22	0.987 (0.615, 1.584)	.955	0.902 (0.562, 1.449)	.670
No	103				
Alcohol drinking					
No	113	0.989 (0.542, 1.804)	.972	1.156 (0.631, 2.116)	.639
Yes	12				
Metastasis					
Yes	100	1.024 (0.637, 1.648)	.921	1.064 (0.661, 1.714)	.798
No	25				
NLR					
Low NLR	71	1.501 (1.033, 2.182)	.033	1.520 (1.043, 2.215)	.029
High NLR	56				

BMI = body mass index, ECOG PS = Eastern Cooperative Oncology Group Performance Status, NLR = neutrophil-to-lymphocyte ratio, PFS = progression-free survival, OS = overall survival.

**Table 4:** The progression-free survival and overall survival in multivariate analyses.

Characteristics	N	PFS		OS	
		HR (95% CI)	*P* value	HR (95% CI)	*P* value
NLR					
Low NLR	52	1.478 (1.015, 2.153)	.042	1.493 (1.022, 2.182)	.038
High NLR	79				

NLR = neutrophil-to-lymphocyte ratio, PFS = progression-free survival, OS = overall survival.

### 3.4. Exploratory analyses of ALC and ANC

**Figure 4.**
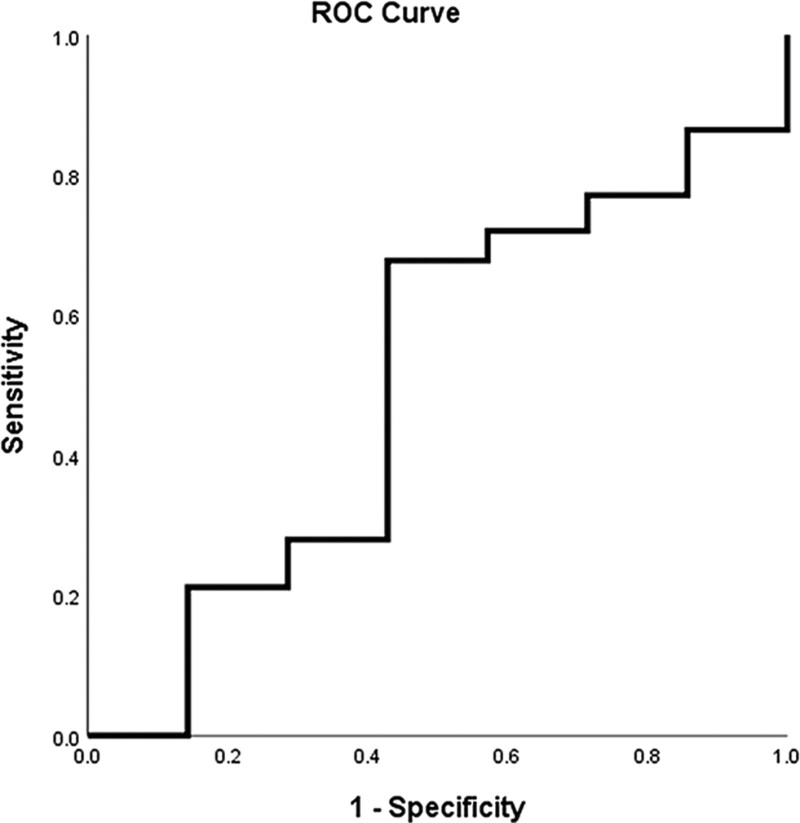
Patients were divided into three groups according to the quantile of absolute lymphocyte count (ALC) and absolute neutrophil count (ANC): progression-free survival (PFS) of ANC (A) and ALC (B); overall survival (OS) of ANC (C) and ALC (D).

To investigate whether the differences in PFS and OS in patients with advanced NSCLC at different levels of NLR are attributable to changes in ALC and ANC, we divided the study population into three groups of these values. The ANC and ALC was not statistically significant regarding PFS and OS in patients with NSCLC (Fig. 4).

## 4. Discussion

In the analysis of 125 patients with advanced NSCLC treated with apatinib after multiline treatment for drug resistance, pretreatment of NLR had a significant independent prognostic factor for this cohort study. Notably, our cohort study population had inferior performance status and was more heavily pretreated (all patients had ≥ 2 prior systemic treatment regimens) compared to clinical trials approving apatinib for solid tumors. Considering these differences, it was understandable that the PFS and OS in our study group were inferior to the published trials. Therefore, this study only represented patients with advanced NSCLC after multi-line drug resistance.

This study explored patients with NSCLC who have progressed after receiving more than two chemotherapy regimens. The NLR status of patients within one month after chemotherapy progression was investigated in this study to determine whether it would affect the anti-tumor efficacy of anti-angiogenic drugs. As for the question of whether the patients’ previous chemotherapy would affect the relationship between the efficacy of anti-VEGF therapy and NLR in this study, it has been shown in the univariate analysis that previous chemotherapy was not the influencing factor in this study. In addition, age, baseline nutritional status, metastasis and ECOG had been proven to be useful biomarkers for the prediction of malignancies, but they also were analyzed in the univariate analysis and were not an important influencing factor in this study. In this observational study, the survival prognosis of patients treated with apatinib was closely related to NLR. After adjusting for gender in the multivariate analysis, NLR was closely related to the efficacy of NSCLC patients resistant to multiple chemotherapy regiments.

In recent years, inflammation in cancer has been a key determinant for the progression and survival of many solid tumor diseases. Many studies have reported resistance to angiogenic drugs in patients with high NLR.^[22,23]^ Anti-VERFR-2 antibodies were used to target tumor blood vessels, resulting in vascular normalization and reprogramming of the immunosuppressive microenvironment to enhance immune activity status. Therefore, vascular normalization through antiangiogenic therapy seems to have important clinical significance for the immune response of tumor cells, especially those mediated by T lymphocyte infiltration.^[24]^ High NLR reflects the severity of systemic inflammation and the decline in tumor-specific immunity. Therefore, we can find changes in peripheral blood neutrophils and lymphocytes to observe the inflammation and immune response in vivo. Currently, it has been studied by scholars as a marker of the impact of the systemic inflammatory response on the survival and prognosis of cancer patients.^[25,26]^ Although it is not clear whether NLR is a predictor in patients with NSCLC treated with apatinib, the marker is easy to use and low cost, which is worthy of further study. We performed survival analysis on the NLR tertiary group and found no statistical significance between the NLR tertiary and patients’ OS and PFS, which proves that NLR = 3.72 as the cutoff value was more suitable for the study population grouping to evaluate the survival prognosis of the study, rather than simply grouping the study population.

Based on this assumption, the study has shown that pretreatment NLR < 3.72 and NLR ≥ 3.72 as cutoff value are associated with OS and PFS. This is consistent with our findings that apatinib had longer PFS and OS in patients with NSCLC with low NLR [high NLR group vs low NLR group between median PFS and OS (1.83 vs 2.76 months, HR 1.478, 95% CI 1.015–2.153, *P* = .042; 2.07 vs 3.40 months, HR 1.493, 95% CI 1.022–2.182, *P* = .038; respectively)]. Compared with the low NLR group and high NLR group, the result showed that the risk of tumor progression increased by 47.8% and the risk of tumor-related death increased by 49.3%.

Nevertheless, the NLR may represent other indicators of the immune state of cancer cells. Considering the antiangiogenic effects of apatinib, in addition to vascular normalization, it also reduces the activity of vascular endothelial growth factor and promotes a multifactor-mediated immune response, which can lead to better survival prognosis. Patients with NSCLC and low NLR are more likely to benefit from apatinib than patients with high one, which can indicate that the microenvironment of NSCLC provides a solid foundation for the effective use of apatinib. In the limited data analysis, no positive correlation was observed between ALC or ANC levels and longer PFS or OS. Therefore, NLR is a better predictor of apatinib effectiveness. As far as we know, the prognostic value of NLR before treatment in patients with NSCLC receiving apatinib after multiline therapy has been reported for the first time.

The cohort study had several limitations. First of all, since the study parameters were derived from clinical medical records and radiological reports, OS and PFS as our primary results were affected by the accuracy of response rate estimates. Second, although we strictly controlled for all hospital inclusion criteria, considering that the study was a single-center retrospective study with small sample size, there was still potential for bias and confusion, such as uncontrolled selection bias. We tried to solve the confounding problem by establishing a multivariate model to adjust confounding factors. Finally, in addition to the clinical trials, patients with NSCLC lacked a routine VEGFR test in clinical treatment. A few patients were tested for indicators such as anti-angiogenic related receptors, which were included in our analysis as potential confounding or interacting variables. Our results were obtained from a homogeneous population of patients with advanced cancer in apatinib therapy after multiline drug resistance treatment and cannot be extrapolated to other populations. Hence, further prospective multicenter studies and a sufficient number of samples are needed to determine the validity of our results.

## 5. Conclusion

In summary, high NLR before treatment is a simple prognostic indicator closely associated with a low survival rate in patients with advanced NSCLC treated with apatinib after multiline treatment for drug resistance. NLR is an inexpensive and clinically available biomarker that provides additional prognostic information to determine which patients would benefit from apatinib treatment.

## Acknowledgments

The authors would like to express their deepest appreciation to all colleagues of the oncology department of Nanhai People’s Hospital for their help in collecting data and proofreading the article. The authors would also like to thank the help of Guangdong Cord blood bank.

## Author contributions

Conception and design: Y.-N.W., H.-M.C., and Y.-Y.L.

Definition of intellectual content: Y.-N.W., Y.-Y.L., and W.-G.G.

Literature search and clinical studies: Y.-N.W., H.-M.C., and X.-F.L.

Data acquisition and analysis: H.-M.C., X.-F.L., and Y.-Y.L.

Statistical analysis: Y.-N.W., H.-M.C., and X.-F.L.

Manuscript preparation, manuscript editing, and manuscript review: All authors.

Final approval of manuscript: All authors.

**Conceptualization:** Ya-Nan Wan, Hai-Ming Chen, Xin-Fu Liu, Wei-Guang Gu, Yi-Yu Lu.

**Data curation:** Ya-Nan Wan, Hai-Ming Chen, Xin-Fu Liu, Yi-Yu Lu.

**Formal analysis:** Ya-Nan Wan, Hai-Ming Chen, Xin-Fu Liu, Wei-Guang Gu.

**Funding acquisition:** Wei-Guang Gu, Yi-Yu Lu.

**Investigation:** Ya-Nan Wan, Hai-Ming Chen, Xin-Fu Liu, Yi-Yu Lu.

**Methodology:** Ya-Nan Wan, Hai-Ming Chen, Xin-Fu Liu, Wei-Guang Gu, Yi-Yu Lu.

**Project administration:** Wei-Guang Gu, Yi-Yu Lu.

**Resources:** Hai-Ming Chen, Wei-Guang Gu, Yi-Yu Lu.

**Software:** Ya-Nan Wan, Hai-Ming Chen, Xin-Fu Liu.

**Supervision:** Hai-Ming Chen, Wei-Guang Gu, Yi-Yu Lu.

**Validation:** Ya-Nan Wan, Hai-Ming Chen, Xin-Fu Liu, Wei-Guang Gu, Yi-Yu Lu.

**Visualization:** Ya-Nan Wan, Hai-Ming Chen, Yi-Yu Lu.

**Writing – original draft:** Ya-Nan Wan, Hai-Ming Chen, Xin-Fu Liu.

**Writing – review & editing:** Hai-Ming Chen, Wei-Guang Gu, Yi-Yu Lu.
